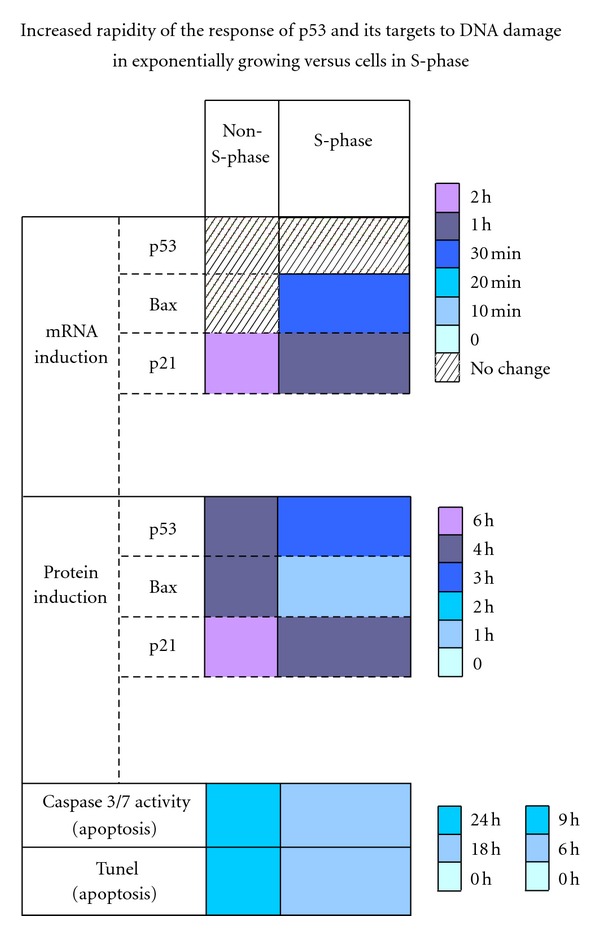# Transcriptional Regulation of the *p53* Tumor Suppressor Gene in S-Phase of the Cell-Cycle and the Cellular Response to DNA Damage

**DOI:** 10.1155/2012/808934

**Published:** 2012-07-11

**Authors:** David Reisman, Paula Takahashi, Amanda Polson, Kristy Boggs

**Affiliations:** ^1^Department of Biological Sciences, University of South Carolina, Columbia, SC 29208, USA; ^2^Departamento de Genética, Faculdade de Medicina de Ribeirão Preto, Universidade de São Paulo, Ribeirão Preto, SP, Brazil; ^3^Department of Hematology-Oncology, St. Jude Children's Research Hospital, Memphis, TN 38105, USA

## Abstract

The *p53* tumor suppressor induces the transcription of genes that negatively regulate progression of the cell cycle in response to DNA damage or other cellular stressors and thus participates in maintaining genome stability. Numerous studies have demonstrated that *p53* transcription is activated before or during early S-phase in cells progressing from G_0_/G_1_ into S-phase through the combined action of two DNA-binding factors RBP-J**κ** and C/EBP**β**-2. Here, we review evidence that this induction occurs to provide available *p53* mRNA in order to prepare the cell for DNA damage in S-phase, this ensuring a rapid response to DNA damage before exiting this stage of the cell cycle.

## 1. Introduction

*p53* is a DNA-binding transcription factor that activates genes responsible for a cell-cycle checkpoint or apoptosis after exposure to ionizing radiation, UV light, or other DNA-damaging agents [1–3]. The *p53* protein is induced both in terms of its abundance and its activity in response to DNA damage. Increased levels of *p53* protein are largely due to increased stability of the protein that is regulated through the loss of association with the MDM2 protein [1–3]. In normal cells where *p53* is found at very low levels, *p53* is present in a complex with MDM2 which targets *p53* for degradation through the ubiquitin pathway [4].

Activation of *p53* has been proposed to occur through a number of mechanisms which include phosphorylation, dephosphorylation by protein serine/threonine phoshatase-1 [5], acetylation by the transcriptional coactivator p300/CBP [6], and induced conformational changes mediated by the prolyl isomerase Pin1 [7–10]. The increase in the level of active *p53* protein leads to an inhibition of entry into S-phase or the induction of apoptosis [2, 11, 12]. Thus, the loss or inactivation of *p53* results in the loss of cell-cycle arrest or apoptosis after DNA damage or physiologic stresses. This loss, seen in many human cancers, has been proposed to lead to increased genetic instability, increased accumulation of mutations, and ultimately oncogenesis.

Interestingly, a number of studies indicate that tumor-derived mutant forms of *p53*, which are highly expressed in many cancers, while losing many of their DNA-damage checkpoint functions, function as active transforming genes [13, 14]. These mutant *p53* genes serve as oncogenes that contribute to tumorigenesis [15–17].

Ever since Arnold Levine's group demonstrated that *p53* expression was induced upon mitogenic stimulation of murine fibroblasts [18], and Reed et al. [19] demonstrated induced expression of *p53* upon mitogenic stimulation of human lymphocytes, and the molecular mechanism responsible for this regulation has remained unexplored. Similarly, an understanding of the biological significance of this induction has remained unclear. This is especially true in light of our current understanding of the role of *p53* as a suppressor of DNA synthesis and inducer of apoptosis. Although elevated levels of *p53* protein have been shown to lead to either growth arrest or apoptosis in response to DNA damage, it might seem anomalous that transcription of the *p53* gene and synthesis of *p53* mRNA are low in cells in G_0_ and are induced upon induction with mitogens, with a peak in transcription prior to DNA synthesis and maximal mRNA synthesis during mid-S-phase. This type of response has been suggested to be important for a rapid *p53*-induced arrest in DNA synthesis in response to DNA damage at a time when cells are synthesizing DNA and thus would be most susceptible to DNA damaging events. In fact, Mosner et al. [20] demonstrated an exceptionally rapid accumulation of active *p53* protein in response to DNA damage in synchronized cells populations in mid-S-phase. In this paper, we summarize recent data that describes the mechanism of cell-cycle regulation of the *p53* gene and the role that this regulation plays in facilitating the DNA damage response during the S-phase of the cell cycle.

## 2. Regulation of *p53* Gene Expression during S-Phase of the Cell Cycle

### 2.1. *p53* Transcription Is Induced during S-Phase

The levels of *p53* mRNA increase substantially prior to S-phase as early as 8 h after serum stimulation and peak at 18 h after serum stimulation [21, 22]. These results are in agreement with earlier publications [18, 20, 23]. *c-myc* mRNA levels are also increased by 3 h after serum stimulation, while no change in the levels of p21 or 14-3-3*σ* mRNA are detected indicating that while the levels of *p53* mRNA is increased, there is no evidence for active *p53* protein being produced.

The 1.7 Kbp murine *p53* promoter has been seen to recapitulate the elevated transcription of the *p53* gene when placed upstream of the luciferase [21]. Eighteen hours after transfection, the cells were maintained in serum-depleted medium for 24 h and then serum stimulated in order to induce S-phase. The 1.7 Kbp promoter decreased in activity after 24 h of serum depletion with a 4-fold reduction in expression and demonstrates an induction of promoter activity with maximal promoter activity after 24 h serum stimulation and entry into S-phase. Analysis of the region required for this induction was ultimately narrowed down to a 20 bp region mapping between −953 and −972 nucleotides upstream of the transcription initiation site [21, 22]. Database searches for transcription factors that may bind the *p53* promoter within this region have provided possible leads as to what protein(s) are binding the promoter within this critical element. Two candidates that have proven to be involved in *p53* regulation are C/EBP*β* and RBP-J*κ* [21, 22].

### 2.2. C/EBP*β*

C/EBP**β** is a CCAAT enhancer-binding protein (35) and is critical for the normal growth and differentiation of various cell types [24–26]. Three protein isoforms of C/EBP**β** are formed by alternative translation of three in-frame initiation sites on C/EBP**β**mRNA [27–29]. C/EBP**β**-1 is the full-length form of the protein (38 KDa) that contains an intact N-terminal transactivation domain and C-terminal DNA-binding domain. C/EBP**β**-2 (35 kDa) differs from C/EBP**β**-1 by only 21 amino acids at the N-terminus; however, the N-terminal transactivation domain is still functional. Both C/EBP**β**-1 and C/EBP**β**-2 are transactivators, although only recently have studies addressed their functional differences. C/EBP**β**-3 (21 kDa) completely lacks the N-terminal transactivation domain and is thought to repress transcription by complexing with C/EBP**β**-1 or =2 and inhibiting their ability to transactivate target genes [27–29].

Electrophoretic mobility shift assays demonstrated binding by endogenous C/EBP**β** to the *p53* promoter [21]. Anti- C/EBP**β** antibody, specific for the C-terminal DNA-binding domain, when included in the DNA-binding assays, resulted in a supershift of the bound complex. A C/EBP**β** neutralizing peptide, which blocks the ability of the C/EBP**β** antibody to bind, prevented the supershift and demonstrates the specificity of the anti-C/EBP**β** antibody. To assay for C/EBP**β** binding the *p53* promoter during the cell cycle, nuclear extracts from arrested and serum-treated Swiss3T3 cells were assayed, and upon growth arrest in G_0_, there was a decrease in C/EBP**β** binding to the promoter. By 3 h after serum and entry into S-phase stimulation, binding of C/EBP**β** increased substantially and coincides with increased endogenous *p53* mRNA levels and an increase in *p53* promoter activity at 3 h after serum stimulation. Finally, it was demonstrated through the use of ChIP assays that C/EBP**β**-2 binding occurs *in vivo* in a manner that is similar to the *in vitro* binding pattern described above [30]. Transfection studies have demonstrated that C/EBP**β**-2 activated expression of the *p53* promoter upon binding to the identified DNA sequence [21].

### 2.3. RBP-J*κ*

RBP-J**κ** is a 60 kDa DNA-binding transcription factor that shows a high degree of conservation across species ranging from *Drosophila* to human [31]. The factor has been shown to be a direct target of the Notch receptor which is central in the regulation of development and differentiation of numerous cell lineages during mammalian development [32–35]. Activation of the Notch receptor results in release of RBP-J**κ** from associated corepressors and the recruitment of coactivators [33, 36, 37]. Thus, in the absence of Notch signaling RBP-J**κ** functions as a transcriptional repressor.

DNA-binding assays performed using nuclear extracts from Swiss3T3 cells that were growing either exponentially, serum depleted for 24 hours, or serum stimulated were employed to test for the presence of RBP-J**κ** by adding an anti-RBP-J**κ** antibody to the binding reaction. Maximal RBP-J**κ**-binding activity is observed after cells are serum starved and arrested in G_0_ [38]. As *p53* mRNA levels start to increase as cells enter S-phase, RBP-J**κ**-binding activity to the regulatory site on *p53* consistently decreases. This supports the findings that RBP- J**κ** acts as a repressor of *p53* transcription, since its binding activity is reduced as cells are released from G_0_. Finally, it was demonstrated through the use of ChIP assays that RBP binding occurs *in vivo* in a manner that is similar to the *in vitro* binding pattern observed. Transfection studies have demonstrated that RBP-J**κ** repressed expression of the *p53* promoter upon binding to the identified DNA sequence [38].

The results indicate that at least two transcriptional regulatory proteins bind to the −972/−953 regulatory region on the *p53* gene and play two very different roles in regulating transcription of this important tumor suppressor. C/EBP**β**-2 serves to enhance *p53* transcription during the transition from the growth-arrested state to the entry into S-phase, while RBP-J**κ** serves to repress *p53* transcription during this transition. These results suggest that both factors (C/EBP*β*-2 and RBP-J**κ**) may work cooperatively or in a coordinated manner to help regulate the activity of *p53* throughout the cell cycle.

## 3. *p53*-Mediated DNA Damage Response in S-Phase

### 3.1. *p53*-Mediated Induction of Bax and p21 in Response to DNA Damage

To investigate the rate of the *p53* DNA-damage response as cells enter S-phase, two *p53* targets, Bax and p21, were evaluated by RT-PCR analysis after treatment of cells with camptothecin, a cytotoxic compound which inhibits the DNA topoisomerase I resulting in DNA damage [39]. These experiments showed that both Bax and p21 mRNA levels were induced by 10 to 18 hrs after camptothecin treatment in exponentially growing cells, while the induction of Bax and p21 mRNA expression in cells in S-phase was exceptionally rapid, occurring within 60–90 minutes and remained 3- to 4-fold higher throughout the experiment.

### 3.2. Binding of *p53* to the Bax Promoter in Response to DNA Damage

Chromatin immunoprecipitation (ChIP) analysis was performed in order to examine the rate of binding of *p53* to the *Bax* promoter in response to DNA damage [39]. Results of a series of ChIP assays demonstrate that in cells not in S-phase and exposed to camptothecin, the binding of *p53* to the Bax promoter remained constant throughout the experiment while in cells entering S-phase, an increase in *p53* binding to the Bax promoter is observed after 0.5 hr of drug exposure and continued to increase over four hours. These findings indicate that, in response to camptothecin treatment, Bax levels in cells entering S-phase are expressed in a more rapid manner than in cells that are not in S-phase. In response to DNA damage in cells in S-phase, *p53* protein levels increase, bind to the *Bax* promoter, and cause a more rapid expression of this proapoptotic regulator.

### 3.3. Apoptosis during S-Phase

The activity of caspases is one useful indicator of apoptosis. Therefore, the activities of two of these proteases, caspases 3 and 7, which are at the end of the apoptotic cascade, were measured in exponentially growing, and in cells entering S-phase after treatment with camptothecin. The activity of caspases 3 and 7 increased between 3 and 6 h in non-S-phase cells but between 0 and 3 h in cells both in S-phase. In addition to increasing more rapidly, the overall activity of these two caspases was higher in cells both in early and late S-phase. These findings indicate that apoptosis is induced earlier and to a greater extent in cells subjected to DNA damage during S-phase, during the period of enhanced *p53* transcription [39].

DNA fragmentation is also a marker of late-stage apoptosis. Cells were treated with camptothecin and subjected to a TUNEL assay that labels the end of DNA fragments in cells undergoing apoptosis. The results of these assays demonstrate that by 18 h after drug exposure, the number of cells in S-phase with fragmented DNA was signifcantly greater compared to the same time point in non-S-phase cells.

## 4. Summary

*p53* induces the transcription of genes that negatively regulate progression of the cell cycle in response to DNA damage or other cellular stressors and thus participates in maintaining genome stability. Under stress conditions, *p53* must be activated to prohibit the replication of cells containing damaged DNA. Numerous studies have demonstrated that *p53* transcription is activated before or during early S-phase in cells progressing from G_0_/G_1_ into S-phase via a coordinated expression of two transcription factors, RBP-J**κ** and C/EBP*β*-2, that act as a repressor and activator of *p53* gene expression, respectively, through their binding to the same site on the promoter. In examining the rates of expression of *p53* target genes and the rates of entry into apoptosis, evidence has accumulated that indicates that this induction occurs to provide sufficient *p53* mRNA to ensure a rapid response to DNA damage before exiting S-phase.  Figure 1 is a summary of published data illustrating the increase in rate of the p53 response when cells are in S-phase.

## Figures and Tables

**Figure 1:**